# Professional Autonomy, Burnout, and Intentions to Leave the Profession Among Spanish Midwives: A Qualitative Study

**DOI:** 10.1155/jonm/3839068

**Published:** 2026-10-20

**Authors:** Rodríguez-Puente Zaida, Pérez-Rivera Francisco Javier, Fernández-Martínez Elena, Liébana-Presa Cristina, González-García Alberto, Andina-Díaz Elena

**Affiliations:** ^1^ Department of Nursing and Physiotherapy Universidad of León León 24004 Spain; ^2^ CECAVIS Research Group IBIOLEÓN León Spain; ^3^ Midwife León Hospital León Spain; ^4^ SALBIS Research Group Universidad de León León Spain unileon.es; ^5^ HeQoL Research Group Universidad de León León Spain unileon.es; ^6^ EYCC Research Group University of Alicante Alicante Spain ua.es

**Keywords:** autonomy, burnout, empowerment, midwifery, qualitative research

## Abstract

**Background:**

The loss of autonomy in midwives is associated with lower job satisfaction and consequently greater likelihood of leaving the profession. Given the international shortage of professionals, it is essential to explore how to increase their job satisfaction and reduce attrition.

**Objective:**

To explore Spanish midwives’ perceptions of the causes of their burnout and reasons for leaving the profession.

**Methods:**

Descriptive qualitative study. Four discussion groups were held with a total of 21 participating midwives between October 10 and 29, 2024. A thematic analysis of the data was performed. The Ethics Committees of the University of León (ETICA‐ULE‐033‐2020) and the University Healthcare Complex of León (Registration Number 20162) approved the study.

**Results:**

A total of 66.7% of the participants were women, 61.9% worked in hospital care, 47.6% were between 30 and 40 years old, and 57.1% had between 10 and 20 years of professional experience. The thematic analysis revealed several main themes: Causes (“Loss of autonomy,” “Excessive medicalization of childbirth,” “Conflict with gynecologists,” “Traumatic perinatal events,” “Lack of representation in management,” and “Working conditions”) Solutions (“Increased professional autonomy,” “Recognition of competences,” “Independent midwifery degree,” and “Professional Recognition”), and Consequences (“Demotivation, Anxiety, and Frustration” and “Leaving the profession”).

**Conclusions:**

In addition to improving overall well‐being within the profession, improving midwives’ mental health can have a positive impact on professional retention, which would help in the significant crisis caused by the international shortage of professionals.

**Implications for Nursing Management:**

Nursing and midwifery managers play a pivotal role in preventing burnout by creating organizational environments that support midwives’ professional autonomy, meaningful participation in leadership and policy decisions, and respectful interprofessional relationships. Management should ensure access to structured debriefing and psychological support following traumatic perinatal events, promote safe staffing and sustainable workloads, and invest in professional development and supportive workplace cultures. These strategies may improve midwives’ wellbeing, strengthen workforce retention, and ultimately enhance the quality and safety of maternity care.

**Summary**
• What is already known?
◦ The significant shortage of midwives is an international problem and it is necessary to explore ways to increase their job satisfaction and reduce attrition from the profession.
• What this paper adds?
◦ Our findings highlight the causes of midwives’ burnout and job dissatisfaction.
◦ Reasons such as lack of autonomy and lack of recognition cause them to be demotivated and to consider leaving the profession.
◦ We suggest that changes be introduced to promote development of midwives’ autonomy and recognition of the profession.

## 1. Introduction

The significant shortage of midwives worldwide has increased interest in studying the reasons for burnout and job dissatisfaction that are related to job turnover [1].

Worldwide, midwives report stress, heavy workloads, scarce resources, and lack of recognition as main drivers of leaving the profession [2, 3].

Midwives face a number of risks, such as long and unpredictable shifts involving nights and other unsociable hours, and experiences of abuse in the workplace from a variety of sources, including patients, family members, colleagues, and hospital management [4].

Organizational factors such as understaffing, low pay, poor management, and toxic environments contribute significantly to burnout [5].

Autonomy, self‐fulfillment, and recognition are central to resilience and satisfaction [6, 7]. Many also attribute their loss of passion for the profession to care pressure, resource shortages, poor communication, and lack of leadership [8].

Studies in the United Kingdom show two‐thirds of midwives have considered leaving the profession [9, 10]. This has far‐reaching costs, as it is associated with sick leave, an intention to leave the job, and consequent higher staff turnover [11].

The main factors contributing to midwives’ resilience are autonomy at work, decision‐making power, self‐management of problems, close communication with colleagues, support from peers and medical staff, a sense of usefulness and responsibility, satisfaction from applying knowledge to practice, working with mothers, and sharing moments of happiness [12, 13]. Their job satisfaction is directly related to factors including autonomy, the importance of the work, the challenges of balancing work and private life, and the high emotional and physical demands placed on them [3, 14]. Promoting autonomy in midwives and recognition is thus vital [15], especially in highly medicalized maternity systems [16].

Midwives’ professional autonomy appears to be particularly limited in many countries, and they also face increasing obstacles to performing their role as legally defined by the directives on the scope of the profession in Europe [17]. Emerging data show the impact of the care model in which midwives work on their well‐being at work [18].

The most recent National Maternity Review in England highlighted that midwives are more likely than other to experience work‐related stress, possibly due to the stressors they face working within a highly medicalized maternity care system. This is deeply concerning, as staff well‐being is a powerful predictor of a positive patient experience, with staff engagement closely linked to quality and safety of care, as well as lower levels of absenteeism [19].

The WHO and ICN (2022; 2025) stress that midwives’ autonomy is essential for maternal and newborn health. Spain faces an acute shortage, with around 8000 registered midwives versus 10,000 qualified [20, 21].

We need to recognize the factors that influence the limitation of midwives’ autonomy, as well as the changes in their professional identity, to understand why they leave the profession [22]. More research with a qualitative approach is needed to allow a deeper exploration of these perspectives. Ours is the first comprehensive qualitative study to examine the factors that affect burnout in midwives in Spain along with their reasons for leaving the profession.

In Spain, the practice of midwives is legally regulated under the official title of Obstetric‐Gynecological Nursing Specialist, requiring a 4‐year Bachelor’s Degree in Nursing followed by a competitive 2‐year specialized residency program via the Nurse Resident in Specialized Training (EIR) pathway [23] (Ministry of Health and Social Policy, 2009). These specialists practice with full technical autonomy across both Primary Care (within community health centers and sexual/reproductive health units) and Specialized Care in hospital environments, encompassing comprehensive care throughout the woman’s life cycle (Ministry of Health and Social Policy, 2009). However, data from the Spanish General Council of Nursing alert to a severe structural shortage that places the midwife‐to‐population ratio significantly below the OECD average, a chronic deficit that exposes the workforce to high workloads, chronic stress, and a heightened risk of burnout [24] (Spanish General Council, 2026). Within this demanding landscape, analyzing the levels of professional empowerment and its relationship with workplace burnout is critical for designing healthcare management strategies that protect midwives’ occupational health and ensure quality of care.

### 1.1. Aim

To explore Spanish midwives’ perceptions of the causes of burnout and reasons for leaving the profession, as well as possible solutions.

## 2. Methods

### 2.1. Study Design

This study employed a descriptive qualitative design to explore midwives’ perceptions and experiences regarding professional autonomy, burnout, and intentions to leave the profession in Spain. A descriptive qualitative approach was considered appropriate because it enables an in‐depth exploration of participants’ perspectives while remaining close to their own accounts, making it particularly suitable for addressing practice‐oriented questions in healthcare. This design allows researchers to generate a rich description of participants’ experiences without imposing the interpretative framework associated with specific qualitative traditions [25]. The study was reported in accordance with the Consolidated Criteria for Reporting Qualitative Research (COREQ) [25].

### 2.2. Participants

Twenty‐one midwives from eight different autonomous communities participated. Participants were selected using a snowball sampling method [26]. The invitation to participate in the study was sent out through the Spanish Federation of Midwives’ Associations (FAME), and midwives who agreed to participate were subsequently contacted by email. Meetings were organized according to participants’ availability (Supporting information 1 and 2). An attempt was made to organize the groups as heterogeneously as possible, including midwives of different genders and ages, from different fields and locations, and with varied years of experience. The only inclusion criterion was to be a certified midwife, and inclusion was progressive and ended with data saturation [27], which was independently evaluated by two researchers when new data were obtained.

### 2.3. Data Collection

Four discussion groups were conducted by three of the researchers, using a list of ad hoc questions based on a previous systematic review that referred to the main themes identified: sociodemographic factors, psychological factors, and occupational factors. The groups were held online via Google Meet between October 10 and 29, 2024.

The participation of 21 midwives from different locations and work environments was considered sufficient for data saturation. The meetings were recorded, and additional notes were taken.

The focus groups were led by one of the researchers, a woman and midwife with healthcare and research experience, and two other researchers, both nurses in qualitative research, also participated as observers.

Before each meeting began, the study objectives were explained to each participant, and their informed consent was requested for participation and recording the meeting.

Participants were sent an invitation to the meeting by email with the details of the meeting and a questionnaire to collect sociodemographic variables. The meetings were held in a quiet office located in the Faculty of Nursing at the University of León.

### 2.4. Data Analysis

The content of each meeting was transcribed, and the data were managed using Microsoft Excel. A thematic approach was used for the analysis. The texts were subjected to multiple complete readings, while the recordings were reviewed simultaneously, with the aim of exploring and understanding the text as well as the different nuanced meanings.

Data were analyzed using Braun and Clarke’s reflexive thematic analysis, following their six‐phase approach to identify, analyze, and interpret patterns of meaning across participants’ narratives. Reflexive thematic analysis was selected because of its methodological flexibility and its suitability for descriptive qualitative research, allowing the research team to develop themes grounded in participants’ accounts while acknowledging the active role of researchers in the interpretative process. [28]. These phases consisted of initial code generation and theme search, coding data characteristics, and collating codes into possible themes and subthemes; reviewing and labeling themes and generating a thematic map of the analysis; and report writing, selecting vivid, compelling, accurate examples, and relating the analysis to the research question and literature. Selected quotes were incorporated into the manuscript to add meaning to the defined themes.

### 2.5. Rigor and Trustworthiness

Trustworthiness was established following the criteria proposed by Lincoln and Guba (1985) [29]. Credibility was enhanced through in‐depth semistructured interviews, data saturation, and independent coding by two researchers, followed by analytical discussions to refine and agree on the final themes. Dependability was ensured through a transparent analytical process following Braun and Clarke’s six‐phase thematic analysis, with analytical decisions documented in an audit trail using Microsoft Excel. Confirmability was supported through regular team discussions, maintenance of a reflexive journal, and continuous comparison of themes with the original transcripts. Transferability was facilitated by providing a detailed description of the study context, participants, and data collection procedures. Participant transcript review (member checking) was not undertaken; instead, trustworthiness was strengthened through researcher reflexivity, collaborative analysis, and the use of illustrative quotations.

The quality recommendations established by Braun and Clarke (2006) [28] were followed. Participants were free to share their perceptions and experiences without restriction, and the authors did not participate by supporting or rejecting any of the opinions, nor did they provide any positive or negative reinforcement [30]. In addition, to ensure the accuracy and consistency of the findings, direct quotes were extracted and several research team meetings were held to reach a consensus on the topics to be defined [31]. A self‐critical attitude was maintained throughout the process. To avoid bias in the formulation of the research questions (given that one of the researchers was a midwife), the questions were developed in accordance with previous studies. To avoid influencing data collection, participant recruitment, and site selection, three of the researchers had only superficial knowledge of the topic (as health professionals) and it was not their usual work/research topic. The researchers began the analysis after the first interview in order to constantly verify that it was in line with the objectives and to be prepared in case any changes needed to be made to the research design. No additional interviews were necessary to clarify the information collected in the groups.

### 2.6. Ethical Considerations

Participants received information about the study and gave their consent both to participate and to have the meetings recorded, with the option to withdraw from the study without negative consequences, in accordance with the Declaration of Helsinki. Furthermore, collection of personal data was not permitted, in accordance with the Ethics Committees of the University of León (ETICA‐ULE‐033‐2020), and the University Healthcare Complex of León (Registration Number 20162) approved the study Supporting information 3 and 4.

### 2.7. Data Availability Statement

The datasets generated and analyzed during the current study are not publicly available due to the sensitive nature of the qualitative interview data and the need to protect participants’ confidentiality. Deidentified data may be available from the corresponding author on reasonable request, subject to ethical approval and institutional requirements.

## 3. Results

### 3.1. Participants

As Table 1 shows, a total of 21 midwives from eight different autonomous communities participated. Seven were men (33.3%) and 14 were women (66.7%). The majority (57.1%) had between 10 and 20 years of experience. A total of 61.9% worked in specialized care, 23.8% in primary care, and 14.3% were university teachers. The 47.6% proportion of the sample was between 30 and 40 years old.

**TABLE 1:** Sample characteristics.

	*N*	%
Gender		
Female	14	(66.7%)
Male	7	(33.3%)
Age range		
30–40	10	(47.6%)
40–49	5	(23.8%)
50–59	5	(23.8%)
> 60	1	(4.8%)
Years of experience		
0–5	1	(4.8%)
5–10	4	(19%)
10–20	12	(57.1%)
> 20	4	(19%)
Workplace setting		
Community	5	(23.8%)
Hospital	13	(61.9%)
University	3	(14.3%)

The results of the midwives’ testimonies were divided into three themes: causes, consequences, and solutions. Within each theme, different categories were identified, as shown in Table 2 and Figure 1.

**TABLE 2:** Coding tree produced by extracting codes and abstracting findings in common categories and themes.

Main theme	Themes	Codes
Causes	Loss of autonomy	
	Excessive medicalization of childbirth	
	Conflict with gynecologysts	
	Traumatic perinatal events	
	Lack of representation in management	
	Working conditions	Job insecurity
		Training
		Salary, retributions
		Family reconciliation

Consequences	Demotivation, anxiety, frustration	
	Leaving the profession	

Solutions	Increased professional autonomy	
	Independent career	
	Professional recognition	Recognition of other professionals
		Recognition of the society

**FIGURE 1:**
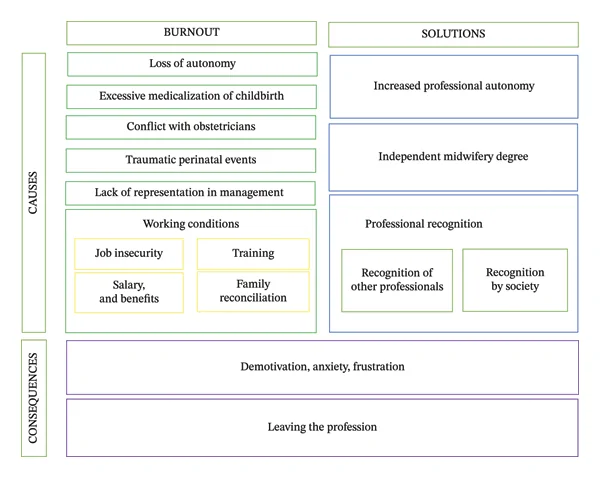
Main theme, themes, and code.

The testimonies and the groups and participants are in the Supporting Information File 1 and Supporting Information File 2.

## 4. Causes

Key burnout causes included loss of autonomy, excessive medicalization, conflicts with obstetricians, traumatic perinatal events, lack of representation in management, and poor working conditions.

Loss of autonomy: Midwives in primary care described greater independence.

“In primary care, you have a lot of independence. A lot. Here, you manage the practice however you want, okay? In that regard, completely. In specialized care, my proposal is that the level of care for births, that is, low‐risk births, should be managed by midwife‐led birth centres, where there are no obstetricians, because they are completely different jobs.”, E7G4

Excessive medicalization of birth was another stressor:

“The situation with inductions: we are talking about delivery rooms that often already have 40%–50%, 40%–50% of inductions, and we are pathologizing and medicalizing many more pregnant women than we should.” E5G1

Another relevant factor influencing midwives’ job dissatisfaction was their working relationship with their obstetrician colleagues.

Many midwives referred to a loss of autonomy in relation to conflict in the hierarchy and power dynamics with their obstetrician colleagues. Many agreed that the medical community sometimes intervenes unnecessarily in normal deliveries, and this influences decision‐making, the development of skills, and independence in the performance of midwives’ professional duties.

“There is a position of dominance of the doctor over the midwife, where decisions are made with a completely vertical hierarchy, and where the degree of autonomy that midwives have is very limited.” E5G3

Traumatic experiences such as stillbirths and obstetric violence caused lasting distress:

“In our work, violent situations arise, whether it be obstetric violence, which is there, or stillborn fetuses, which also cause professional trauma, but the reality is that people endure the strain of their profession and continue to work hard despite their trauma.” E5G2

The lack of representation of midwives in management positions was also described as one of the causes of burnout. Midwives also reported limited support from nonspecialist managers:

“We are not 100% nurses, but we are managed by generalist nurses, and this sometimes makes management complicated and sometimes makes communication and understanding with management difficult, and I think that ultimately ends up burning us out. How many midwives have decision‐making power at the political level? Very few, if any.” E5G3

In terms of working conditions, job instability, lack of training, salary, and difficulty balancing work and family life were some of the causes interviewees cited most often as causes of burnout and exhaustion. A salary that is inadequate to the level of responsibility was one of the causes of dissatisfaction that all participants agreed on.

“Overqualification is not accompanied by a salary commensurate with the responsibilities we ultimately have. Salary and working hours are not in line with responsibility, training, or the reality we live in. Why would I want that responsibility and autonomy if autonomy comes with responsibility and I am paid the same?” E3G4

They also mentioned the importance they placed on their roles as mothers, which can cause their professional careers to take a back seat during a significant period of their lives.

“It is very difficult to **balance family life and work**, and I believe that midwives, as a general rule, are professionals who value parenting highly and who modify our careers by requesting part‐time work, as in my case, where I currently work part‐time to care for my children or am on leave… And sometimes you put your professional career, your motivation, or your promotion on hold, of course. I had a paradigm shift when I became a mother.” E3G2

## 5. Consequences

The consequences of job burnout for midwives included anxiety, frustration, and demotivation, and consequently, leaving the profession.

“People who become midwives are usually very talented and highly motivated. In other words, it’s not easy to demotivate a midwife, but I think that burnout in the profession can often be caused by trying to achieve improvements for the profession and for our skills as a group, and never actually achieving them. In other words, I think that midwifery is a very mistreated profession.” E2G3

It is also important to note that almost half of the midwives interviewed had considered leaving the profession or had left it.

“I gave up. I lost hope. I stopped believing in what I was doing. I worked in primary care and in hospitals, and it was my life’s passion. I felt that the institution didn’t support me.” E2G1.

“I also want to leave when I can, because working like this is really frustrating. It makes you feel incredibly powerless and completely disconnects you from who we are, with the wonderful profession we have”. E1G1

## 6. Solutions

The solution to reducing burnout among midwives involved increasing their level of professional autonomy, which they said requires greater empowerment as a group and the acquisition of higher‐level skills.

They also referred to the possibility of creating midwife‐led birthing centers, as in other neighboring countries, where midwives report feeling autonomous and empowered.

“In order to have more autonomy and empower ourselves as professionals, midwives need to change their mindset and try to take on more of the things that are really ours.” E2G2

Many of the interviewees proposed the solution of having an independent degree course for the profession and disassociating themselves from nursing, ceasing to be a nursing specialty in order to gain autonomy and independence. They believed that this would help gain autonomy and recognition.

“Having an independent degree would help a lot. Being part of nursing means diluting our professional identity. We have a problem with professional recognition, and the healthcare profession has been simplified to doctors and nurses, which sometimes makes it unclear what we are. I do believe that we should have an independent profession to gain autonomy and a well‐defined profession. Healthcare here is very focused on medical care. We need professional recognition, and that starts with having our own, well‐defined profession.” E4G1.

Some of the participants, however, expressed doubts about this, unsure whether it would help the profession.

“I’m not very much in favour of the midwifery degree, and this is a bit unpopular, but that’s how I feel, because I think it’s true that it could solve problems, but I think it would worsen training. The training system is good. In fact, I’ve worked with midwives from all the teaching units in Spain, and I feel admiration for them.” E3G3

One of the solutions that interviewees proposed for tackling occupational burnout and the loss of professionals was to promote recognition of the profession by other professionals and by society.

“There is a lack of recognition, for example, feeling valued by our supervisors, by our management, by our colleagues or even by the medical profession”. E3G1

## 7. Discussion

The objectives of the study were achieved after exploring the causes, consequences, and solutions to midwife burnout.

## 8. Causes

The results of our study describe several causes of burnout that were categorized into several themes: “Loss of autonomy,” “Excessive medicalization of childbirth,” “Conflict with gynecologists,” “Traumatic perinatal events,” “Lack of representation in management,” and “Working conditions” (Figure 2).

**FIGURE 2:**
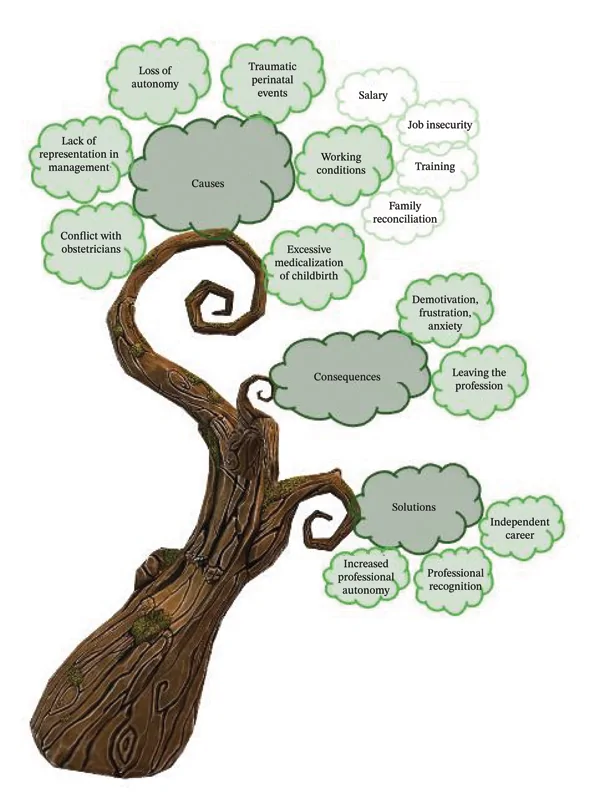
Causes, consequences, and solutions about burnout in midwives.

### 8.1. Loss of Autonomy

The study confirms multifactorial causes of burnout similar to international findings. Loss of autonomy mirrors the impact of hierarchical hospital structures [16]. In the study in South Africa by Matlala et al., 2019 [29], midwives raised concerns that their independent role was ignored by physicians and emphasized that most cesarean sections could perhaps be avoided if midwives were given autonomy.

Primary care midwives generally feel more autonomous and satisfied [16, 32, 33], and they feel more satisfied in using their skills to a greater extent, they tend to experience greater autonomy in the organization of their work, and they are also more satisfied with their work and work–life balance [34, 35].

### 8.2. Excessive Medicalization of Childbirth

Medicalization restricts physiological birth practices and physiological birth has been shown to result in better perinatal outcomes, and midwives play an important role in advocating for physiological birth as noted in Shorey and Debby’s review [36].

The study of Harvie et al., 2019, stated how the medicalization of childbirth and high levels of medical interventionism made them feel “overridden” and prevented them from doing their work autonomously [10]. Similarly, Bloxsome et al., 2019, noted that midwives placed great value on the relationships created with women during the physiological birth process and they felt proud of their ability to promote normality in childbirth, which increased their empowerment and reinforced their desire to remain in the profession [34].

Midwives in New Zealand and Sweden exhibit a deeply rooted professional identity and a higher degree of autonomy, which is probably the result of working more independently and autonomously with a significant and highly structured midwifery network in a cultural context that conceives of childbirth as a life event [10].

### 8.3. Conflict With Obstetricians

Some midwives described were dissatisfaction with the working relationships with their obstetric colleagues. Some stated that midwives’ autonomy is not possible when there are historical hierarchies and power dynamics. Interprofessional collaboration is only possible when professionals respect each other and are in equal positions.

A WHO report confirms that midwives around the world want to be more autonomous, which means that they would need to be given appropriate professional recognition by obstetricians [22]. In Slovenia, for example, obstetricians do not recognize midwives as autonomous professionals for normal pregnancy, childbirth, or postpartum [19].

Participants have also described how unequal power dynamics sometimes contributed to bullying and harassment within practices, which negatively affected midwives’ mental health and they sometimes reported a sense of being undervalued by physicians and managers [4, 37]. Midwives in Flanders and the Netherlands recently indicated the need for a shift towards shared responsibility and autonomy according to Mestdagh et al., 2019 [32].

### 8.4. Traumatic Perinatal Events

Another of the most common causes of job burnout among respondents was adverse perinatal events.

The latest NHS (UK) staff survey showed that almost half of midwives have witnessed stillbirths, neonatal deaths, and brain injuries occurring in childbirth that contributed to staff stress and fatigue [19].

The majority of the midwives interviewed in another study had experienced traumatic births, which significantly impacted their mental health as they relived distressing situations over and over again. Lack of support or time off after these events was identified as having a negative impact on mental health, in addition to a lack of understanding from friends and family about what they had experienced. They also generally described the nature of labor as emotionally draining [4].

More individual or group mental health support seems necessary for them to cope with traumatic events [18]. A consistent recommendation from the Doherty & O’Brien, 2022, a study was to seek a time to reflect with colleagues after an adverse event or stressful workday [38].

### 8.5. Lack of Representation in Management

Health management approaches and difficult interaction with administration and managers may be one of the biggest barriers to empowerment and job satisfaction regardless of where and how they work. Midwives in our study felt frustrated, referring to their superiors not knowing how they worked, and not giving them enough support to develop their skills.

Midwives’ underrepresentation in management limits empowerment and they felt that managers lacked knowledge of midwives’ work context and felt excluded from policy formulation and changes made to their work [37].

Midwives working in organizations with good leadership were less likely to report burnout [39].

### 8.6. Working Conditions

Midwives demonstrated dissatisfaction and discomfort with various factors related to working conditions, such as job instability, lack of training, salary, and the difficulty reconciling work with family life. The pressure of care due to the shortage of midwives, midwife/patient ratios below worldwide recommendations, and precarious contracts produced significant job burnout in midwives that sometimes even led them to consider leaving the profession.

Other studies confirmed the enormous pressure midwives feel, with staff shortages, unmanageable workloads, and the frustration of not being able to provide quality care contributing to a perceived lack of confidence and frequently presented as a source of dissatisfaction [9]. They defined persistent stress and burnout linked to excessive workload related to constant staff shortages [40, 41].

The midwives in our study also noted how important work–life balance was to them and highlighted the importance they place on parenting, which in many cases leads them to take leave or reduced working hours for long periods of time, taking them away from their professional career and opportunities for growth and promotion.

In one previous study, the ability to work part‐time was of great importance to midwives and allowed them the work–life balance they needed. Some midwives felt fortunate to have shift patterns that allowed them to parent with flexibility [33].

The majority of the respondents in the study by Darling et al., 2023, felt that their work–life balance, on‐call schedule, and inability to get enough sleep negatively affected their mental health [4].

None of the midwives in our study felt positive about their salaries and reported feeling “overqualified” for the remuneration they received.

While in the study by Cull et al., 2020, salary did not appear to be a strong motivator for most participants, several respondents did note the importance of access to training, opportunity, and support for career progression [9]. However, in Darling et al., 2023, midwives mentioned being undervalued and poor remuneration in comparison to the emotional effort involved in their work [4]. In addition, the study by Matlala et al., 2019, emphasized the need to be paid accordingly based on qualifications and experience [37].

When it came to training, some participants in our study complained about the difficulties in attending courses and conferences during working hours, a lack of support from the administration, and the lack of funding for training. Other studies have also shown that midwives feel unprepared for some of the nonclinical aspects of their work before starting their careers [4]. The midwives in the study by Matlala et al., 2019, expressed concern about not having the opportunity to attend workshops and symposia and mentioned the importance of training and keeping up to date in their work [37].

## 9. Consequences

The consequences were categorized in two main groups: “Demotivation, Anxiety, and Frustration” and “Leaving the profession.”

### 9.1. Demotivation, Anxiety, and Frustration

The midwives in our study stated that the consequence of all these factors is that they felt stressed, exhausted, frustrated, and poorly motivated. This is in line with findings reported in the United Kingdom, where numerous participants showed emotional distress, exhaustion, depression, stress, and anxiety, describing feeling out of control, unable to enjoy their free time, and shut down [9].

Shorey et al., 2023, analyzed factors influencing the physiological birth process and documented midwives’ struggles to work independently in obstetrician‐led inpatient units that made them feel stressed, frustrated, challenged, and distrustful, while limiting their ability to promote normal physiological birth. In contrast, in “birth centers” or home settings, they felt they had complete autonomy and responsibility, which allowed them to closely monitor labor in a personalized manner [36].

### 9.2. Leaving the Profession

Although it is noteworthy that the midwives interviewed for our study demonstrated a strong passion for their profession, almost half of the interviewees reported that they had considered leaving the profession, and some had indeed left. Leaving the nursing profession, especially among midwives, is a worldwide problem [41, 42].

Hildingsson et al.’s, 2023, results showed that approximately 30% of Swedish midwives considered leaving the profession and their reasons are in line with our results: lack of staff and resources, high levels of stress, work conflicts, low pay, and concern for their personal health [22].

Almost half of the midwives in other Australian study indicated that they had considered leaving the profession in the previous 6 months. The two most common reasons for wanting to leave were “dissatisfaction with the organization” and “dissatisfaction with my role as a midwife.” The midwives felt that they were continually serving the needs of the organization rather than the women [10].

Other studies have discussed the testimonies of some midwives expressing uncertainty about the long‐term sustainability of the profession, mentioning the high attrition rate of midwives and commenting on how the rigidity of the model contributes to them leaving the profession [4].

However, other midwives we interviewed did not consider leaving the profession because of the passion they felt for their work. This is consistent with studies where midwives define the profession as stressful but state that the good days somehow justify staying in practice [34], while others state that despite the challenges, they feel fulfilled in their profession, describing it as a source of pride and self‐esteem [9]. Other midwives have described their love and passion for their profession and said that they felt privileged to provide care to women and their families [10]. And in the study by Matlala et al., 2019, despite feeling demoralized by obstacles related to their daily work environment, midwives also expressed their passion for the profession and hence their intention to remain in it [37].

Leadership strategies and working conditions influence the retention, satisfaction, and professional roles of midwives within healthcare teams [43, 44].

## 10. Solutions

This category includes “Increased professional autonomy,” “Recognition of competences”, “Independent midwifery degree,” and “Professional Recognition.”

### 10.1. Increased Professional Autonomy

The factor most frequently mentioned by the midwives in our study as a cause of job burnout was the loss of autonomy, and many of them called for an increase in autonomy as a solution to the loss of personnel and to increase their motivation and satisfaction. This is consistent with several studies where interviews have shown that midwives define their ability to be autonomous and in control of their own practice as something that made them feel more confident. Many study participants enjoyed facilitating natural childbirth by working autonomously and highly valued the positive feedback from women and their families. They also mentioned how much they appreciated and valued the autonomy of midwife‐led continuity models [4, 9]. Sabzevari et al., 2019, noted that job autonomy increases midwives’ resilience [13].

Salary, autonomy, and passion for the profession were other factors that explained why midwives chose to remain in the profession [34].

Recent WHO guidelines emphasize that midwives should work autonomously within interdisciplinary teams to ensure quality care and call for global expansion of midwifery models of care [45, 46].

The WHO promotes midwife‐led continuity of care models because evidence demonstrates that these models improve maternal outcomes and satisfaction, and this is linked to a high degree of professional autonomy within these models [46, 47].

### 10.2. Independent Midwifery Degree

One of the solutions the participating midwives proposed was the development of a separate degree to nursing and although no published articles have been found that refer to midwives’ opinions on this matter, we did find some reference to this issue in an article by Harvie et al., 2019, in Australia [10]. It noted that some midwives stated that they sometimes feel frustrated and unable to develop their competencies as midwives and some of the testimonies reflected the following: “I feel like a nurse midwife rather than a midwife.”

### 10.3. Professional Recognition

Another solution proposed was to encourage recognition of the profession by peers and society. In Belgium, where interviewed, midwives highlighted their desire to be recognized and respected by society and other health professionals [16].

Midwives in the study by Darling et al., 2023, felt that some patients did not have a good understanding of midwives’ profession, their role, or the scope of their practice, and this negatively impacted their mental health. They also felt that other health professionals did not understand their profession well [4]. The need for support and recognition was a resounding theme identified in the meetings in the study by Doherty and O’Brien, 2022 [40].

Recognition by the medical profession is significant for the profession, as can be seen in different studies where midwives feel poorly recognized by their colleagues in the medical profession [8] and the lack of recognition from the medical profession was one of the main reasons related to job burnout [22].

In Sweden, like other countries, midwives are leaving the profession due to the stressful work environment, medicalization of birth, and lack of professional recognition [22].

### 10.4. Limitations

The study has some limitations and the findings should be considered with caution. Although there was representation of midwives from different settings, both rural and urban, most worked in specialized care, and although there was representation of different genders in each group, most of the interviewees were women.

The interviews were conducted online, and in these cases, it is harder to establish a rapport than in a face‐to‐face interview. Social desirability (giving “politically correct” answers) may also have played a role.

In contrast, one of the strengths of the study is that it provides comprehensive qualitative data in response to quantitative data obtained in previous studies. There is also a large research gap in the field. Future research could introduce a more heterogeneous sample and setting.

One interesting future line of research would be to compare these results with other midwifery focus groups from different countries and examine the differences.

### 10.5. Implications for Nursing Management

Burnout among midwives should be recognized as an organizational workforce and quality‐of‐care issue rather than solely an individual resilience problem. The findings of this study have important implications for nursing and midwifery management.

Nurse and midwifery managers should prioritize strategies that strengthen midwives’ professional autonomy by supporting evidence‐based, midwife‐led models of care and ensuring that midwives are able to practice to the full scope of their competencies. Increasing midwife representation in leadership, management, and policy‐making structures may also improve decision‐making processes, professional empowerment, and organizational commitment.

The findings further highlight the importance of fostering respectful interprofessional collaboration between midwives, obstetricians, and other maternity care professionals. Leadership initiatives aimed at reducing hierarchical barriers, promoting shared decision‐making, and improving mutual professional recognition may contribute to healthier workplace cultures and lower levels of burnout.

Managers should also implement organizational support systems following traumatic perinatal events, including structured debriefing sessions, access to psychological support services, and opportunities for peer support and clinical supervision. These interventions may help reduce the long‐term emotional impact of adverse events and strengthen workforce resilience.

Finally, healthcare organizations should address modifiable workplace factors such as inadequate staffing, excessive workloads, limited opportunities for professional development, and difficulties achieving work–life balance. Investing in supportive leadership, safe staffing, and healthy work environments is likely to improve job satisfaction, workforce retention, and the quality and safety of maternity care.

## 11. Conclusions

This descriptive qualitative study is the first comprehensive investigation of midwives’ perceptions of the factors that impact their mental health and burnout in Spain. Our analysis identified the main factors that negatively impact their mental health. These include a loss of professional autonomy, overmedicalization of childbirth, conflict with gynecologists as a group, traumatic perinatal events, under‐representation of midwives in management positions, and negative working conditions.

The impact of these factors on midwives has led to demotivation, anxiety, frustration, and some cases leaving the profession.

The solutions proposed in our study include increasing midwives’ autonomy, recognition of the profession, and the creation of an independent degree qualification.

In addition to improving overall wellbeing within the profession, improving midwives’ mental health may have a positive impact on retention, which would help in the major staffing crisis in the profession internationally.

Fostering healthy relationships and teamwork between midwives and others and improving respect and recognition of the profession are areas that require innovative interventions and multifaceted approaches.

## Funding

This research received no specific grant from any funding agency in the public, commercial, or not‐for‐profit sectors.

## Conflicts of Interest

The authors declare no conflicts of interest.

## Supporting Information

Additional supporting information can be found online in the Supporting Information section.

## Supporting information


**Supporting Information 1** FILE 1: Main Themes, Themes, and Codes and Quotes.

**Supporting Information 2** FILE 2: Table showing the distribution of interviewees in each discussion group.

**Supporting Information 3** FILE 3: Invitation to participate in the study and link to the questionnaire.

**Supporting Information 4** FILE 4: Informed consent.

## Data Availability

The data that support the findings of this study are available from the corresponding author upon reasonable request.

## References

[1] Cramer E. and Hunter B. , Relationships Between Working Conditions and Emotional Wellbeing in Midwives, Women and Birth. (2019) 32, no. 6, 521–532, 10.1016/j.wombi.2018.11.010. 30578019

[2] Catling C. , Donovan H. , Phipps H. , Dale S. , and Chang S. , Group Clinical Supervision for Midwives and Burnout: A Cluster Randomized Controlled Trial, BMC Pregnancy and Childbirth. (2022) 22, no. 1, 10.1186/s12884-022-04657-4. PMC8999988 35410189

[3] Hansson M. , Dencker A. , Lundgren I. , Carlsson I. M. , Eriksson M. , and Hensing G. , Job Satisfaction in Midwives and Its Association With Organisational and Psychosocial Factors at Work: A Nation-Wide, Cross-Sectional Study, BMC Health Services Research. (2022) 22, no. 1, 10.1186/s12913-022-07852-3. PMC8976984 35366877

[4] Darling E. K. , Grenier L. N. , MacKenzie R. K. et al., A Mixed-Method Study Exploring Barriers and Facilitators to Midwives’ Mental Health in Ontario, BMC Women’s Health. (2023) 23, no. 1, 10.1186/s12905-023-02309-z. PMC10066979 37005669

[5] Suleiman-Martos N. , Albendín-García L. , Gómez-Urquiza J. L. et al., Prevalence and Predictors of Burnout in Midwives: A Systematic Review and Meta-Analysis, International Journal of Environmental Research and Public Health. (2020) 17, no. 2, 10.3390/ijerph17020641. PMC7013833 31963831

[6] Jiang X. M. , Chen X. Q. , Zheng J. , Zheng Q. X. , Huang X. X. , and Pan Y. Q. , A Multi-Center Cross-Sectional Study on Midwives’ Self-Efficacy and Influencing Factors, Japan Journal of Nursing Science. (2020) 17, no. 4, 10.1111/jjns.12357. 32588969

[7] Nutor J. J. , Aborigo R. A. , Okiring J. et al., Individual and Situational Predictors of Psychological and Physiological Stress and Burnout Among Maternity Providers in Northern Ghana, PLoS One. (2022) 17, no. 12, 10.1371/journal.pone.0278457. PMC9754239 36520845

[8] Catling C. and Rossiter C. , Midwifery Workplace Culture in Australia: A National Survey of Midwives, Women and Birth. (2020) 33, no. 5, 464–472, 10.1016/j.wombi.2019.09.008. 31676324

[9] Cull J. , Hunter B. , Henley J. , Fenwick J. , and Sidebotham M. , Overwhelmed and out of My Depth: Responses From Early Career Midwives in the United Kingdom to the Work, Health and Emotional Lives of Midwives Study, Women and Birth. (2020) 33, no. 6, e549–e557, 10.1016/j.wombi.2020.01.003. 31980392

[10] Harvie K. , Sidebotham M. , and Fenwick J. , Australian Midwives’ Intentions to Leave the Profession and the Reasons Why, Women and Birth. (2019) 32, no. 6, e584–e593, 10.1016/j.wombi.2019.01.001. 30635229

[11] Matthews R. , Hyde R. , Llewelyn F. , Shafiei T. , Newton M. , and Forster D. A. , Factors Associated With Midwives’ Job Satisfaction and Experience of Work: A Cross-Sectional Survey of Midwives in a Tertiary Maternity Hospital in Melbourne, Australia, Women and Birth. (2022) 35, no. 2, e153–e162, 10.1016/j.wombi.2021.03.012. 33935006

[12] Doering K. , McAra-Couper J. , and Gilkison A. , Attuning to the World of Peace of Mind and Trust: Women’s Lived Experience of the Woman–Midwife Relationship in Japan, Qualitative Health Research. (2024) 34, no. 10, 964–976, 10.1177/10497323241226568. 38365201

[13] Sabzevari M. T. and Rad M. , Resilience Strategies Against Working Pressures in Midwives: A Qualitative Study, Journal of Education and Health Promotion. (2019) 8, no. 1, 10.4103/jehp.jehp_241_18. PMC6432840 30993126

[14] Wangler S. , Streffing J. , Simon A. , Meyer G. , and Ayerle G. M. , Measuring Job Satisfaction of Midwives: A Scoping Review, PLoS One. (2022) 17, no. 10, 10.1371/journal.pone.0275327. PMC9560034 36228002

[15] Janighorban M. , Dadkhahtehrani T. , Najimi A. , and Hafezi S. , The Correlation Between Psychological Empowerment and Job Burnout in Midwives Working in the Labor Ward of Hospitals, Iranian Journal of Nursing and Midwifery Research. (2020) 25, no. 2, 128–133, 10.4103/ijnmr.IJNMR_100_19. PMC7055180 32195158

[16] Vermeulen J. , Fobelets M. , Fleming V. , Luyben A. , Stas L. , and Buyl R. , How Do Midwives View Their Professional Autonomy, Now and in Future?, Healthcare. (2023) 11, no. 12, 10.3390/healthcare11121800. PMC10297976 37372919

[17] Mivšek P. A. , Hundley V. , van Teijlingen E. , Pahor M. , and Hlebec V. , Slovenian Midwifery Professionalization: Perception of Midwives and Related Health Professions, Eur J Midwifery. (2021) 5, no. July, 30–10, 10.18332/ejm/137664. PMC8290859 34316548

[18] Stoll K. and Gallagher J. , A Survey of Burnout and Intentions to Leave the Profession Among Western Canadian Midwives, Women and Birth. (2019) 32, no. 4, e441–e449, 10.1016/j.wombi.2018.10.002. 30341004

[19] England NHS , National Maternity Review. Better Births: Improving Outcomes of Maternity Services in England, 2017, NHS England, London.

[20] World Health Organization (WHO) , Midwives Voices, Midwives Realities: Findings From a Global Consultation on Providing Quality Midwifery Care, 2022, WHO, Geneva, https://apps.who.int/iris/handle/10665/250376, [cited 2022]. Available from.

[21] Fokeladeh H. S. , Montayre J. , and Stewart D. , International Council of Nurses 2025 Congress: Nursing Power to Change the World, International Nursing Review. (2024) 71, no. 4, 681–683, 10.1111/inr.13077. 39601325

[22] Hildingsson I. , Fahlbeck H. , Larsson B. , and Johansson M. , How Midwives’ Perceptions of Work Empowerment Have Changed Over Time: A Swedish Comparative Study, Midwifery. (2023) 118, 10.1016/j.midw.2023.103599. 36657224

[23] Ministry of Health and Social Policy , Order SAS/1349/2009 of May 6, Approving and Publishing the Training Programme for the Specialty of Obstetric-Gynaecological Nursing (Midwifery), Boletín Oficial del Estado. (2009) no. 129, 45155–45184, https://www.boe.es/diario_boe/txt.php?id=BOE-A-2009-8881, Available from.

[24] General Council of Nursing , Spain Needs 10,000 More Midwives to Safeguard Women’s Health, 2026, General Council of Nursing, Madrid, Spain, https://www.consejogeneralenfermeria.org, [cited 2026 Jun 23]. Available from.

[25] Braun V. and Victoria C. , Is Thematic Analysis Used Well in Health Psychology? A Critical Review of Published Research, with Recommendations for Quality Practice and Reporting, Health Psychology Review. (2023) 17, no. 4, 695–718, 10.1080/17437199.2022.2161594. 36656762

[26] Kaplan C. D. , Korf D. , and Sterk C. , Temporal and Social Contexts of Heroin-Using Populations: An Illustration of the Snowball Sampling Technique, The Journal of Nervous and Mental Disease. (1987) 175, no. 9, 566–574, 10.1097/00005053-198709000-00009. 3655783

[27] Vasileiou K. , Barnett J. , Thorpe S. , and Young T. , Characterising and Justifying Sample Size Sufficiency in Interview-Based Studies: Systematic Analysis of Qualitative Health Research Over a 15-Year Period, BMC Medical Research Methodology. (2018) 18, no. 1, 10.1186/s12874-018-0594-7. PMC6249736 30463515

[28] Braun V. and Clarke V. , Thematic Analysis: A Practical Guide, 2021, Sage, 1–100.

[29] Saunders B. , Sim J. , Kingstone T. et al., Saturation in Qualitative Research: Exploring Its Conceptualization and Operationalization, Quality and Quantity. (2018) 52, no. 4, 1893–1908, 10.1007/s11135-017-0574-8. PMC5993836 29937585

[30] Schwandt T. A. , Lincoln Y. S. , and Guba E. G. , Judging Interpretations: but Is It Rigorous? Trustworthiness and Authenticity in Naturalistic Evaluation, New Directions for Evaluation. (2007) 2007.

[31] Morse J. M. , Critical Analysis of Strategies for Determining Rigor in Qualitative Inquiry, Qualitative Health Research. (2015) 25, no. 9, 1212–1222, 10.1177/1049732315588501. 26184336

[32] Mestdagh E. , Timmermans O. , Fontein-Kuipers Y. , and Van Rompaey B. , Proactive Behaviour in Midwifery Practice: A Qualitative Overview Based on Midwives’ Perspectives, Sexual & Reproductive HealthCare. (2019) 20, 87–92, 10.1016/j.srhc.2019.04.002. 31084826

[33] Weltens M. , de Nooijer J. , and Nieuwenhuijze M. J. , Influencing Factors in Midwives’ Decision-Making During Childbirth: A Qualitative Study in the Netherlands, Women and Birth. (2019) 32, no. 2, e197–e203, 10.1016/j.wombi.2018.06.009. 29935989

[34] Bloxsome D. , Ireson D. , Doleman G. , and Bayes S. , Factors Associated With Midwives’ Job Satisfaction and Intention to Stay in the Profession: An Integrative Review, Journal of Clinical Nursing. (2019) 28, no. 3–4, 386–399, 10.1111/jocn.14651. 30129076

[35] Clemons J. H. , Gilkison A. , Mharapara T. L. , Dixon L. , and McAra-Couper J. , Midwifery Job Autonomy in New Zealand: I Do It All the Time, Women and Birth. (2021) 34, no. 1, 30–37, 10.1016/j.wombi.2020.09.004. 32962945

[36] Shorey S. and Ng E. D. , Midwives’ Perceptions of and Experiences With Normal Physiologic Birth: A Qualitative Systematic Review, Birth. (2023) 50, no. 4, 749–763, 10.1111/birt.12763. 37712184

[37] Matlala M. S. and Lumadi T. G. , Perceptions of Midwives on Shortage and Retention of Staff at a Public Hospital in Tshwane District, Curationis. (2019) 42, no. 1, e1–e10, 10.4102/curationis.v42i1.1952. PMC6676782 31368315

[38] Doherty J. and O’Brien D. , A Participatory Action Research Study Exploring Midwives’ Understandings of the Concept of Burnout in Ireland, Women and Birth. (2022) 35, no. 2, e163–e171, 10.1016/j.wombi.2021.06.002. 34130937

[39] Albendín-García L. , Suleiman-Martos N. , Cañadas-De la Fuente G. A. , Ramírez-Baena L. , Gómez-Urquiza J. L. , and De la Fuente-Solana E. I. , Prevalence, Related Factors, and Levels of Burnout Among Midwives: A Systematic Review, J Midwifery Womens Health. (2021) 66, no. 1, 24–44, 10.1111/jmwh.13186. 33463008

[40] Doherty J. and O’Brien D. , Reducing Midwife Burnout at Organisational Level – Midwives Need Time, Space and a Positive Work-Place Culture, Women and Birth. (2022) 35, no. 6, e563–e572, 10.1016/j.wombi.2022.02.003. 35181238

[41] Doherty J. and O’Brien D. D. , Giving of the Self and Midwife Burnout: An Exploration of the Consequences of Being ‘With Woman’ and How Individual Midwives Can Reduce or Prevent Burnout, Women and Birth. (2023) 36, no. 4, 349–356, 10.1016/j.wombi.2022.12.002. 36526542

[42] Marufu T. C. , Collins A. , Vargas L. , Gillespie L. , and Almghairbi D. , Factors Influencing Retention Among Hospital Nurses: Systematic Review, British Journal of Nursing. (2021) 30, no. 5, 302–308, 10.12968/bjon.2021.30.5.302. 33733849

[43] Doleman G. , Bloxsome D. , Wang R. , and Whitehead L. , Fostering a Resilient Healthcare System: Supportive Practice Environment Initiatives to Support Nurse and Midwives a Scoping Review, Journal of Nursing Management. (2025) 2025, no. 1, 10.1155/jonm/8898411. PMC11968167 40223894

[44] Arundell F. , Mannix J. , Sheehan A. , and Peters K. , Workplace Culture and the Practice Experience of Midwifery Students: A Meta-Synthesis, Journal of Nursing Management. (2018) 26, no. 3, 302–313, 10.1111/jonm.12548. 29076580

[45] World Health Organization , Implementation Guidance on Transitioning to Midwifery Models of Care, 2025, World Health Organization.

[46] Hopkins B. , WHO Calls for Global Expansion of Midwifery Models of Care, Neonatology Today. (2025) 20, no. 6.

[47] Sandall J. , Soltani H. , Gates S. , Shennan A. , Devane D. et al., Midwife‐Led Continuity Models Versus Other Models of Care for Childbearing Women, Cochrane Database of Systematic Reviews. (2015) 9. 10.1002/14651858.CD004667.pub4 26370160

