# Supplementary material for: Professional Autonomy, Burnout, and Intentions to Leave the Profession Among Spanish Midwives: A Qualitative Study
Source: J Nurs Manag. 2026 Oct 20;2026:3839068. doi: 10.1155/jonm/3839068 (PMC13681738; doi:10.1155/jonm/3839068)
Supplement: Supplementary file 1 [file JONM-2026-3839068-s001.docx]

**Supplementary material 4.** Informed consent

Good morning,

Regarding the research we are conducting on the lack of autonomy in midwives internationally, we are contacting you to invite you to participate in a focus group to address a qualitative research on midwives.

We are in a time of international midwifery shortage, and the review of the scientific literature shows how the lack of autonomy and empowerment is directly affecting burnout, burnout and attrition in midwives. The aim of the research is to identify factors that contribute to greater empowerment among Spanish midwives and to inform strategies to improve job satisfaction and retention in the profession.

If you agree to participate, we would like to know your availability to conduct the meeting, which will be held telematically through the Google Meet platform. It will be recorded for data analysis, after your consent, and subsequently deleted after the end of the research.

Please reply to this e-mail confirming if you would like to participate and check the option below that best suits you:

Morning schedule/ Afternoon schedule.

Time zone:

We thank you in advance for your attention and invaluable help in the development of this study.

The data obtained will be used exclusively by the researchers. These data will be used in accordance with the General Data Protection Regulation and the Organic Law 3/2018 on the Protection of Personal Data and Guarantee of Digital Rights. If you wish to receive information about the purpose and procedure of the study, as well as the general results of the research, you can request it through the following email [zrodp@unileon.es](mailto:zrodp@unileon.es).
