# Supplementary material for: Professional Autonomy, Burnout, and Intentions to Leave the Profession Among Spanish Midwives: A Qualitative Study
Source: J Nurs Manag. 2026 Oct 20;2026:3839068. doi: 10.1155/jonm/3839068 (PMC13681738; doi:10.1155/jonm/3839068)
Supplement: Supplementary file 2 [file JONM-2026-3839068-s002.docx]

**Supplementary Material File 1:** Main Themes, Themes and Codes and Quotes

| Main Theme | Themes | Codes | Example of Quotes |
| --- | --- | --- | --- |
| Causes | Loss of autonomy |  | *“Our autonomy depends largely on the extent to which an external professional, which is the gynecologist in this case, allows or does not allow us to do our work. We are still quite far behind in terms of our ability to work independently and autonomously because we need permission from the gynecologist.”. E3G2* |
|  | Excesive medicalization of childbirth |  | *“The situation of inductions, we are talking about delivery rooms many times with 40-50, 40-50% of inductions and we are pathologizing and medicalizing many more pregnant women than we should be.”. E5G1* |
|  | Conflict with obstetricians |  | *“There is a position of dominance of the doctor over the midwife, where decisions are made in a totally vertical hierarchy, and where the degree of autonomy that midwives have is very limited.”. E5G3* |
|  | Traumatic perinatal events |  | *“En nuestro trabajo se dan situaciones violentas, ya sea por la violencia obstétrica, que está ahí, como en los fetos muertos, que sí que van creando heridas profesionales también, pero la realidad es que la gente aguanta aguanta el tirón de su profesión y sigue y sigue al pie del cañón con sus heridas”. E5G2* |
|  | Lack of representation in management |  | *“In our work there are violent situations, either because of obstetric violence, which is there, or because of dead fetuses, which do create professional wounds as well, but the reality is that people endure the pull of their profession and go on and on with their wounds.”. E5G3* |
|  | Working conditions | Job insecurity | *“We are a very temporary staff, very punished. As soon as they send you to primary care or to specialized care, they change your service... I am almost 43 years old and I am still an interim, that also demotivates, you try to go to other provinces, or to do things. The basis is to make longer contracts, more dignified and without a lot of excess hours, because we are exposed to a lot of emotional burden.”.* |
|  |  | Tranining | *“Tenemos la dificultad de la super-especialización.. Nuestros compañeros, por ejemplo, del Reino Unido, en eso sí que están un poquito más puestos a punto, en el que las matronas se pueden super-especializar en duelo perinatal, en medidas alternativas de alivio del dolor, en parto de bajo riesgo... Y aquí, sin embargo, o te lo buscas tú, o no se motiva al especialista a seguir formándose”. E4G3* |
|  |  | Salary, Retributions | *“We have the difficulty of super-specialization.... Our colleagues, for example, in the United Kingdom, for example, are a little bit more up to speed, where midwives can super-specialize in perinatal bereavement, in alternative pain relief measures, in low-risk childbirth.... And here, however, either you have to look for it yourself, or the specialist is not motivated to continue training.”E3G4* |
|  |  | Family reconciliation | *“It is very difficult to reconcile family life and work, and in addition, midwives I think that, as a general rule, we are professionals who do have a lot of esteem for parenting and that we modify our career by asking for half days, as is my case, I am currently on a half day for childcare or leave.... And sometimes you pause your professional career, your motivation or your promotion as a matter of course. I have had a paradigm shift when I became a mother”. E2G4* |
| Consequences | Demotivation, Anxiety, Frustration |  | *“I mean, it is not easy to demotivate a midwife, but the burnout of the profession, I think, can often be trying to achieve improvements for the profession and for our competencies, as a collective, and we never get there. In other words, I think that the midwifery profession is a very abused profession”E2G3,* |
|  | Leaving the profession |  | *“I gave up. I lost the illusion. I stopped believing in what I was doing. I have worked in primary care, in hospital and it was my life's passion. I felt that the institution did not support me”E2G1.*  *“I also want to leave when I can, because working like this is really frustrating, it makes you incredibly impotent and totally disconnects you from what we are, with the wonderful profession we have.”. E1G1*  *“I have thought about it many times, about quitting. The thing is that what reconciles me with the profession is women”.E3G1*  *“I am one of those midwives who is on the verge of giving up, I was happier at work as a nurse than I am now, now every day I have to fight for the woman.” E4G2*  *“I have not found my place. I have been harassed by many fellow midwives and gynecologists because they did not understand my way of working. Simply because I used to close the door and ask them to call me to come in for an expulsion. I was on sick leave for five months, also with a lot of anxiety and depression. And finally I had to make the decision, after 10 years occupying a vacancy I resigned, I left, not knowing where I was going to end up. I really don't know if I will ever go back to a delivery room, but I think my place is not there because I lose my autonomy, I lose working with my own values. And to the extent that I can't apply my values, or what I think is good to accompany, I feel like I'm just working according to medical indications”E3G2.*  *“I am on maternity leave and I am thinking of taking a leave of absence because I can't stand working in this system. I am seriously considering changing profession, I love the profession of midwife and there is nothing I like more in the world, I love to accompany women, and that has left me sleepless, but come on, I mean, of course I have to change the direction of my life because the truth is that it is a nightmare to be like this.”E5G4.* |
| Solutions | Independent career |  | *“The fact that we were an independent career would help a lot. Being within nursing means diluting our professional identity. We have a problem of professional recognition and the health profession has been simplified to doctors and nurses in which sometimes we are not very clear about what we are. I do believe that we should have an independent profession to gain autonomy and a well-defined profession. Healthcare here is very focused on medical care. We need professional recognition and that starts with having our own defined profession”E4G1.* |
|  | Professional recognition | Recognition of other professionals | *“There is a lack of recognition, for example, feeling valued by our supervisors, by our management, by our colleagues or even by the medical profession of which you have spoken so much.”.E3G1* |
|  |  | Recognition of the society | *“The change has to be social and I believe and hope that in a few years we will have the social recognition we need to have. I am 31 years old and I have friends who did not know that we attended births or that we accompanied the woman throughout the birth process. My mother did not know either, I think that social pressure is what will make us be recognized, as in England and in these countries that are much more advanced, where midwives have always been at the forefront of physiological childbirth and the skills of a midwife as a midwife”. E1G4* |
