# Supplementary material for: Professional Autonomy, Burnout, and Intentions to Leave the Profession Among Spanish Midwives: A Qualitative Study
Source: J Nurs Manag. 2026 Oct 20;2026:3839068. doi: 10.1155/jonm/3839068 (PMC13681738; doi:10.1155/jonm/3839068)
Supplement: Supplementary file 3 [file JONM-2026-3839068-s003.docx]

**Supplementary material 2.** Table showing the distribution of interviewees in each discussion group

| Interviewee | Gender | Age Range | Years Experience | Workplace setting |
| --- | --- | --- | --- | --- |
| E1G1 | Female | 30-40 | 10-20 | AE |
| E2G1 | Female | 50-59 | 10-20 | AE |
| E3G1 | Male | >60 | >20 | AP |
| E4G1 | Female | 30-40 | 10-20 | AE |
| E5G1 | Female | 50-59 | >20 | AE |
| E6G1 | Female | 30-40 | 10-20 | AE |
| E7G1 | Female | 30-40 | 10-20 | AP |
| E1G2 | Male | 30-40 | 10-20 | Uni |
| E2G2 | Female | 40-49 | 10-20 | AE |
| E3G2 | Male | 50-59 | 10-20 | AE |
| E4G2 | Male | 40-49 | 5-10 | AE |
| E5G2 | Female | 30-40 | 10-20 | AE |
| E1G3 | Female | 40-49 | 10-20 | AE |
| E2G3 | Male | 40-49 | >20 | Uni |
| E4G3 | Female | 30-40 | 5-10 | AE |
| E5G3 | Male | 40-49 | 10-20 | AP |
| E6G3 | Male | 30-40 | 5-10 | Uni |
| E1G4 | Female | 30-40 | 0-5 | AP |
| E1G4 | Female | 50-59 | 10-20 | AE |
| E2G4 | Female | 50-59 | >20 | AP |
| E3G4 | Female | 30-40 | 5-10 | AE |
