# Supplementary material for: Professional Autonomy, Burnout, and Intentions to Leave the Profession Among Spanish Midwives: A Qualitative Study
Source: J Nurs Manag. 2026 Oct 20;2026:3839068. doi: 10.1155/jonm/3839068 (PMC13681738; doi:10.1155/jonm/3839068)
Supplement: Supplementary file 4 [file JONM-2026-3839068-s004.docx]

**Supplementary material 3.** Invitation to participate in the study and link to the questionnaire

We are in a time of international midwifery shortage, and the review of the scientific literature shows how the lack of autonomy and empowerment is directly affecting midwifery burnout, attrition and attrition.

A positive relationship has been described between midwives' empowerment and their desire to remain in the profession. Other factors closely related to empowerment have also been identified, such as receiving support from superiors, having access to sufficient resources, or perceiving self-determination and independence in their work, which contribute positively to the desire to remain in the profession.

This questionnaire is part of a research, led by Dr. Francisco J. Pérez Rivera and Dr. Elena Andina Díaz (Department of Nursing and Physiotherapy and SALBIS research group, University of León, Spain), PhD student Zaida Rodríguez Puente (Department of Nursing and Physiotherapy, University of León, Spain) and Dr. Olga Arias Gundín (Department of Psychology, Sociology and Philosophy, and CAEL research group, University of León, Spain).

The objective of the research is to identify factors that contribute to greater empowerment among Spanish midwives and to inform strategies to improve job satisfaction and retention in the profession.

The most widely used instrument to measure the perceived empowerment of midwives is the Perceptions of Empowerment in Midwifery Scale (PEMS), which has been validated in Spain, we ask for your collaboration and consent by participating in the study by answering the following questions which will take very few minutes.

We thank you in advance for your attention and invaluable help in the development of this study.

The data obtained will be used exclusively by the researchers. These data will be used in accordance with the General Data Protection Regulation and the Organic Law 3/2018 on Personal Data Protection and Guarantee of Digital Rights. If you wish to receive information about the purpose and procedure of the study, as well as the general results of the research, you can request it through the following email zrodp@unileon.es.

<https://docs.google.com/forms/d/1RXV0cvzImQw8AvNsgQ63JVY3jStechxc1a7fkiHdjU/viewform?edit_requested=true#responses>
